# Childhood Underinsurance in Primary Care: A Practice-Based Study

**DOI:** 10.3390/healthcare13192427

**Published:** 2025-09-25

**Authors:** Brooklynne A. S. Dilley-Maltenfort, Samantha A. Roberts, Serena K. Kaul, Caroline M. Goeller, Adrienne Stolfi, Gregory Eberhart, Katherine M. Perry, John M. Pascoe

**Affiliations:** 1 Department of Pediatrics, Wright State University Boonshoft School of Medicine, Dayton, OH 45435, USA; dilley.7@wright.edu (B.A.S.D.-M.); kaul.4@wright.edu (S.K.K.); goeller.9@wright.edu (C.M.G.); adrienne.stolfi@wright.edu (A.S.); gregeberhart@gmail.com (G.E.); perry.224@wright.edu (K.M.P.); 2 Dayton Children’s Hospital, 1 Children’s Plaza, Dayton, OH 45404, USA

**Keywords:** child health, cross-sectional studies, insurance coverage, Ohio, medically underinsured, surveys and questionnaires, population health, COVID-19, primary care, Affordable Care Act

## Abstract

**Background/Objectives:** Health insurance coverage is critical for children’s health, yet underinsurance remains a significant issue in the United States. This study aims to estimate the prevalence and correlates of childhood underinsurance in southwestern Ohio during a portion of the COVID-19 pandemic. **Methods:** This is a cross-sectional study of a convenience sample of children, ages 6 months to <18 years, seen in primary care pediatric practices. Recruitment of children’s primary caregivers (PCGs) occurred in practice waiting rooms from June 2021 to April 2023. Respondents completed the Medical Expenses of Children Survey (MEoCS). Index children were considered underinsured if their PCG responded “yes” to any of six questions regarding the inability to pay for a child health clinician’s recommendation despite the child having health insurance. Chi-squared tests and logistic regression were employed in data analysis. **Results:** 1252 PCGs completed the MEoCS with a response rate of about 90%. 11.3% of index children were underinsured. 41.5% of PCGs raising underinsured children found it harder to access care for their child compared to 3 years ago, while only 9.5% of PCGs raising adequately insured children reported it was harder (*p* < 0.001). PCGs of underinsured children were more likely to report that COVID-19 had a negative effect on their household income (49.2%) and their child’s school performance (52.0%) and mental health (47.7%) compared to adequately insured children (27.0%, 27.0%, 25.0%; *p* < 0.001). **Conclusions:** About 1 in 9 index children were underinsured. Lower parental education and private health insurance were associated with underinsurance across several study cohorts, documenting the stability of these drivers of underinsurance.

## 1. Introduction

Health insurance coverage for children is critical for their health, yet underinsurance remains a significant issue in the United States [1]. Approximately one in three privately insured children and one in eight publicly insured children experience underinsurance, and children with medical complexity are particularly vulnerable. Among privately insured children with multiple chronic conditions, 42% are underinsured. The primary driver of underinsurance among privately insured children is affordability. Almost two-thirds (60%) of families raising children with private health insurance report having unreasonable out-of-pocket costs. This is compared to only 16% of families raising children with public insurance [2]. A major challenge in addressing childhood underinsurance is the lack of a universal definition. This complicates cross-study comparisons and hinders the development of targeted policy interventions. Daw et al. analyzed the National Survey of Children’s Health (NSCH) data from 2016 to 2021 and defined underinsurance as coverage that failed to meet one or more of the following criteria for children ages 0 through 17: consistently meeting their healthcare needs, reliably providing access to necessary healthcare providers, and either having no annual out-of-pocket expenses or keeping such costs at a reasonable level [2].

Similarly, Yu et al., using the NSCH data from 2016 to 2019, defined childhood underinsurance according to the Title V Maternal and Child Health Services Block Grant Program’s National Performance Measure (NPM) 15. This measure considers children adequately insured if they have continuous coverage that meets their medical needs, provides access to required services and clinicians, and reasonably covers associated costs. Their analysis found an increase in underinsured U.S. children from 30.6% in 2016 to 34% in 2019, largely driven by rising out-of-pocket expenses. Children most likely to be underinsured include those with private insurance, those with increased health complexity, and those living in households with a family income below 400% of the federal poverty level [3]. Children who are underinsured often live in families who cannot afford clinician-recommended healthcare despite having insurance coverage for their children [3,4,5,6].

While childhood underinsurance is more prevalent than uninsurance, gaps in coverage remain a concern. In 2023, 5.8% of U.S. children were uninsured [7]. Compared to underinsured children, uninsured children face even greater barriers to healthcare access, fewer preventive visits, and increased financial strain on families [8]. Children and youth with special healthcare needs (CYSHCN) are particularly vulnerable to both insurance inadequacy (e.g., underinsurance) and coverage disruptions (e.g., periods of uninsurance), exacerbating unmet healthcare needs [3,9]. Gigli and Graaf found that ineligibility for services among CYSHCN declined from 2019 to 2020, likely due to pandemic-era policies that expanded access [10]. However, as these temporary policies have been reversed, coverage losses have increased, particularly following the unwinding of continuous Medicaid enrollment [11]. This trend highlights the urgent need for policy interventions that not only prevent uninsurance but also enhance the adequacy of coverage among insured children, ensuring consistent access to necessary care.

Child health policymakers and researchers are concerned about the erosion of the initial progress achieved by the policies enacted in the 1980s and, more recently, the Affordable Care Act due to families’ declining participation in Medicaid and/or the Children’s Health Insurance Program since 2016 [3,12]. Using data from the 2016 to 2019 NSCH and defining underinsurance as continuous coverage with inadequate coverage of needed medical services, Gaffney et al. found that both uninsurance and underinsurance increased nationally, with the proportion of underinsured children rising from 22.8% in 2016 to 25.4% in 2019. Millions of underinsured children had chronic health conditions, highlighting the serious implications of inadequate coverage [9].

Childhood underinsurance exacerbates disparities in healthcare access and “casts a long shadow into their adulthood”, [13] leading to higher rates of preventable conditions and hospitalizations in adulthood, ultimately placing a greater burden on healthcare systems. Ensuring comprehensive insurance coverage for children is a critical step to reduce disparities across the life cycle and improve population health [14,15].

Population health encompasses the overall health status of a defined group, including the distribution of health outcomes within that population [16]. These outcomes are influenced by a complex interplay of factors such as healthcare access, genetics, socioeconomic conditions, environmental exposures, and policies that shape health trajectories across a lifetime [17]. Addressing these determinants collectively is essential to improve population health. This study focuses on one key determinant—childhood health insurance coverage.

This study expands upon an earlier study in southwestern Ohio that documented ongoing childhood underinsurance post-Affordable Care Act [5,18]. Earlier studies identified lower parental education, lower family income, private insurance, and poorer child health as factors associated with higher underinsurance rates [5]. This study estimates the prevalence of and factors related to underinsurance among index children seen in pediatric offices within a primary care practice-based research network in southwestern Ohio. This project continues to estimate the prevalence of underinsurance among index children following the implementation of the Affordable Care Act and to identify correlates of underinsurance during a portion of the COVID-19 pandemic.

## 2. Materials and Methods

### 2.1. Materials

The survey employed in this study, the Medical Expenses of Children Survey (MEoCS), has been utilized in similar research since 2009 [5,18]. It is an adaptation of the University of Colorado’s High Plains Research Network Community Council validated Voorhees’ Medical Expenses Survey [6], that utilized seven questions to determine whether respondents were underinsured. The colonoscopy screening question was deemed irrelevant for children, and it was omitted. However, the remaining six questions were included in this study, and the response options remained unchanged (yes, no, and don’t know). This written survey was self-administered, anonymous, and voluntary. To determine the index child’s underinsurance status, primary caregivers were asked if, in the last 12 months, due to inability to pay, they (1) delayed care for their child, (2) were unable to make or keep an appointment for their child, or they were unable to (3) take their child to see a specialist, (4) obtain a test, (5) fill a prescription, or (6) get other medical care for their child that a clinician recommended. A child was classified as underinsured if their primary caregiver responded “yes” to 1 or more of these questions. The survey assessed primary caregivers’ and index children’s demographics, the index child’s health insurance coverage over the past 12 months, and the source of insurance coverage (private or public). In addition, the survey included questions about whether the child’s health had been adversely affected due to the inability to pay for needed medical care and the ease of obtaining care for the child now compared to 3 years ago. The survey also assessed the impact of the COVID-19 pandemic on household income, and index children’s mental health and school performance. The study was approved by the Wright State University and Dayton Children’s Hospital Institutional Review Boards.

### 2.2. Study Sites

The Southwestern Ohio Ambulatory Research Network (SOAR-Net) is a primary care practice-based research network created in 2002. It includes 9 practices located in economically and geographically diverse areas of the Miami Valley of Ohio. This study was conducted at 9 SOAR-Net practices, and all participating practices agreed to a minimum of 1 week of recruitment time.

### 2.3. Methods

Primary caregivers of index children were recruited in the waiting rooms of participating practices. Primary caregivers were eligible if they were literate in English and the child they accompanied was at least 6 months and less than 18 years old. A research assistant was available at all times to answer respondents’ questions. Respondents included birth parents, grandparents, adoptive parents, or foster parents. If multiple children were seen at one appointment, primary caregivers were asked to complete the survey using the youngest child as the subject. Following a preliminary pilot study with 30 questionnaires, data collection for the study was conducted during the later stages of the COVID-19 pandemic, beginning in June 2021 and continuing through April 2023. An identical questionnaire was employed throughout the duration of the study.

### 2.4. Statistical Analysis

1252 participants completed the study questionnaire, and the response rate was about 90%. Surveys were not included in the analysis if: the respondent was not the primary caregiver, the child’s age was not provided or was outside the study range, the child’s insurance status was not provided, the child was uninsured, or if responses to two or more of the six underinsurance questions were missing or marked as “Don’t know.” The analyses were performed on the remaining 1174 surveys.

Descriptive statistics included mean and standard deviation (SD) for continuous variables and frequency (percent of non-missing responses) for categorical variables. Categorical variables were analyzed using chi-square or Fisher’s exact tests to determine unadjusted associations among independent variables and underinsurance status. Multiple logistic regression analysis was performed to calculate the adjusted odds ratios and 95% confidence intervals for factors associated with underinsurance status. Children who were adequately insured were used as the reference group for multiple logistic regression analysis. The multiple logistic regression analysis included independent variables associated with underinsurance in univariate analyses. All analyses were conducted with IBM SPSS Statistics for Windows, v29.0 (IBM Corporation, Armonk, NY, USA). *p* values < 0.05 were considered statistically significant.

## 3. Results

The majority of study respondents were white (75.6%) and were married or part of an unmarried couple (66.9%). Almost all respondents (94.7%) were parents of the index child, and 76.6% were their mothers. The mean (SD) age for index children was 7.5 (5.1) years, and the mean (SD) respondent age was 37.2 (9.1) years. About half of the index children were male (52.2%). Over half (55.1%) of respondents reported an annual household income of $50,000 or more, and 40.3% had a bachelor’s degree or higher. Just over half (52.6%) of the index children had private health insurance coverage, and almost all had continuous coverage over the past 12 months (96.6%). Per the Ohio Children’s Hospital Foundation, about one half of children in Ohio have public health insurance, so this sample over samples for children with private insurance [19]. Based on the 2023 census estimates, the sample is a reasonably representative cross-section of families residing in Montgomery County, Ohio, with racial demographics closely matching those reported in the census [20].

As noted above, index children were classified as underinsured if primary caregivers reported an inability to obtain clinician-recommended care for their child based on a “Yes” response to one or more of the six questions of the MEoCS. About 1 in 9 index children (11.3%) were classified as underinsured (Table 1). The most common “Yes” response was to the question regarding the inability to obtain a recommended prescription for their child due to financial difficulties (Table 2).

The age sub-group with the highest rate of underinsurance was index children 5 to 11.9 years old (15.8%). Underinsured index children were more likely to have experienced changes to their insurance in the last 12 months (7.3%) compared to children who were adequately insured (2.9%, *p* = 0.015; Table 1).

Almost a third (30.3%) of primary caregivers of underinsured index children reported their child’s health suffered because of the inability to afford care, compared to less than 1% (0.3%) of primary caregivers of adequately insured index children (*p* < 0.001). More primary caregivers of underinsured children also reported that getting care was harder (41.5%) now versus three years ago compared to primary caregivers of children who were adequately insured (9.5%, *p* < 0.001). Primary caregivers of underinsured children were more likely to report the COVID-19 pandemic negatively affected their household income (49.2%) and their child’s school performance (52%) and mental health (47.7%) compared to primary caregivers of children adequately insured (27.0%, *p* < 0.001; 27.0%, *p* < 0.001; 25.0%, *p* < 0.001; Table 3).

Table 4 summarizes adjusted odds ratios (AOR) and 95% confidence intervals (CI) for the underinsurance outcome. Odds ratios were adjusted for all the variables within the table. The children’s age sub-group and overall health were NOT associated with childhood underinsurance status. Primary caregivers with an associate degree or less were more likely to have an underinsured child than primary caregivers with a college degree or higher (AOR 3.69, 95% CI 2.05–6.62). Children with private health insurance coverage were more likely to be underinsured compared to children with public health insurance coverage (AOR 2.18, 95% CI 1.28–3.69). Primary caregivers who reported that their child’s health suffered in the past 12 months due to the inability to pay for medical care and that getting needed medical care for their child was harder than 3 years ago were more likely to report their child was underinsured when compared to their respective reference groups (AOR 150.77, 95% CI 40.55–560.58; AOR 5.99, 95% CI 3.58–10.03).

## 4. Discussion

This study builds upon previous research by the same investigators published in 2021 that examined the prevalence and correlates of childhood underinsurance following the Affordable Care Act. The 2021 paper reported no difference in the childhood underinsurance rate pre- and post-Affordable Care Act [5]. The current study, conducted during the later stages of the COVID-19 pandemic, utilized a nascent convenience sample of children recruited from the same practice-based research network in the Miami Valley of Ohio (Southwestern Ohio Ambulatory Research Network-SOAR-Net).

Despite the evolution of healthcare policy in this country, lower parental education (defined as less than a bachelor’s degree) and private insurance coverage were consistently associated with underinsurance across all three southwestern Ohio cohorts [5]. The investigators speculate the stability of these variables suggests that these factors are key drivers of childhood underinsurance.

Several studies have found that lower parental education level is associated with lower health insurance literacy, leading to difficulty navigating the health insurance system [21,22,23,24]. In addition, both Daw et al. and Yu et al. found that the increase in the proportion of children facing unreasonable out-of-pocket medical expenses was the main driver behind the rise in insurance inadequacy [2,3].

Sufficient health insurance coverage for affordable access to necessary services and healthcare professionals has been linked to a decrease in unmet healthcare needs and improved quality of care [3,4]. The advantages of childhood health insurance continue into adulthood, leading to reduced hospitalization rates, lower occurrence of chronic conditions, and decreased likelihood of obesity [14,15]. The Families First Coronavirus Response Act (FFCRA) mandated continuous Medicaid enrollment for almost all Medicaid recipients from 18 March 2020, until March 2023 [25]. The termination of this policy may decrease the number of publicly insured children and foster another rise in underinsurance rates. Future studies should examine the sequelae of terminating these policies on childhood uninsured and underinsured rates and children’s health.

The authors suggest that ensuring children have access to comprehensive, high-quality health insurance is a crucial strategy to mitigate disparities, in part because it facilitates timely preventive care, reduces unmet healthcare needs, and improves long-term health trajectories. Thus, addressing childhood underinsurance is not only a matter of individual well-being but also a critical strategy for enhancing population health. Future policy efforts should focus on ensuring comprehensive coverage that guarantees affordable and meaningful access to care. The authors speculate strategies such as expanding Medicaid-CHIP eligibility, reducing out-of-pocket costs through enhanced subsidies, and strengthening benefit requirements for private plans may help address these challenges and promote more equitable healthcare access.

This study has several limitations. As a cross-sectional analysis of a community-based convenience sample of primary caregivers and their children, it is subject to the usual constraints regarding causality [26]. In addition, study participants were recruited at primary care pediatric offices. Thus, primary caregivers in the community who were NOT seeking healthcare for their children at the time of survey collection were excluded. A community-based random sample from southwestern Ohio may have had a higher prevalence of childhood underinsurance than reported in this study [2,3,9]. In addition, the lack of a widely accepted definition of underinsurance makes the comparison of underinsurance rates among studies fraught with difficulties.

Despite these limitations, study data represent more than 1000 families in southwestern Ohio and corroborates earlier research. This study contributes important novel information regarding childhood underinsurance rates and factors related to childhood underinsurance during the later stages of the COVID-19 pandemic. Future longitudinal research should focus on the ongoing effects of the pandemic and childhood underinsurance on population health.

## 5. Conclusions

Adequate health insurance is a critical factor for children’s wellbeing, yet children’s underinsurance in the United States remains an ongoing barrier to children’s good health. Over 10% of the children in this study were underinsured during the 12 months prior to survey collection. Based on this study and earlier work, the investigators speculate that private health insurance and lower parental education are major drivers of childhood underinsurance at this time in our nation’s history.

## Figures and Tables

**Table 1:** Demographic Characteristics.

	Adequately Insured	Underinsured	
Variable/Level	No. (%)	No. (%)	*p* Value
Respondent’s education status (n = 1170)			
≥College graduate	438 (92.8)	34 (7.2)	**<0.001**
≤AA/some college	600 (86.0)	98 (14.0)	
Child’s age category (n = 1174)			
0.0–4.9 years	418 (91.7)	38 (8.3)	**0.001**
5.0–11.9 years	369 (84.2)	69 (15.8)	
12.0–17.9 years	254 (90.7)	26 (9.3)	
Household Income (n = 1146)			
≥$50,000	577 (91.3)	55 (8.7)	**0.002**
<$50,000	439 (85.4)	75 (14.6)	
Child’s overall health (n = 1166)			
Excellent	457 (91.6)	42 (8.4)	**0.011**
Very good	392 (88.1)	53 (11.9)	
Good	153 (85.0)	27 (15.0)	
Fair/Poor	33 (78.6)	9 (21.4)	
Child’s insurance changed in the last 12 months (n = 1129)			
No	977 (89.6)	114 (10.4)	**0.017**
Yes	29 (76.3)	9 (23.7)	
Child’s race (n = 1158)			
White	701 (89.5)	82 (10.5)	0.197
Black	142 (89.3)	17 (10.7)	
Other/Multiple	184 (85.2)	32 (14.8)	
Respondent’s marital status (n = 1169)			
Married/unmarried couple	699 (89.4)	83 (10.6)	0.298
Single/separated/divorced/widowed	338 (87.3)	49 (12.7)	
Respondent’s relation to child (n = 1174)			
Parent	985 (88.6)	127 (11.4)	0.673
Grandparent/other	56 (90.3)	6 (9.7)	
Child’s insurance (n = 1174)			
Public	491 (88.3)	65 (11.7)	0.711
Private	550 (89.0)	68 (11.0)	
Child’s sex (n = 1168)			
Male	541 (88.7)	69 (11.3)	0.932
Female	494 (88.5)	64 (11.5)	
	Mean (SD)	Mean (SD)	*p* Value
Index child’s age (n = 1174)	7.5 (5.2)	7.9 (4.5)	0.162
Respondent’s age (n = 1151)	37.3 (9.3)	36.5 (6.9)	0.768

Bolded *p* values for statistical significance (*p* < 0.05). AA, Associates of Arts; SD, standard deviation.

**Table 2:** Number (%) of “Yes” Responses to Questions Assessing Underinsured Status.

	All Respondents
Underinsurance Status Question	No. (%)
In the last 12 months, due to financial difficulties:	
Were you unable to fill a recommended prescription for your child? (n = 1169)	64 (5.5)
Were you unable to make/keep a regular doctor’s appointment for your child? (n = 1173)	46 (3.9)
Were you unable to take your child to see a referred specialist? (n = 1170)	43 (3.7)
Were you unable to get other needed medical care for your child? (n = 1169)	41 (3.5)
Were you unable to have your child get a recommended test done? (n = 1170)	28 (2.4)
Did you delay seeking medical care for your child? (n = 1166)	61 (5.2)

**Table 3:** Factors Associated with Underinsurance.

	All Respondents	Adequately Insured	Underinsured	
Variable/Level	No. (%)	No. (%)	No. (%)	*p* Value
In the past 12 months, did your child’s health suffer because of the inability to pay for medical care? (n = 1172)				
No	1129 (96.3)	1037 (99.7)	92 (69.7)	**<0.001**
Yes	43 (3.7)	3 (0.3)	40 (30.3)	
How has the ease of getting medical care your child needs changed compared to 3 years ago? (n = 1063)				
Easier/Stayed the same	924 (86.9)	855 (90.5)	69 (58.5)	**<0.001**
Harder	139 (13.1)	90 (9.5)	49 (41.5)	
What effect did the COVID-19 pandemic have on your income? (n = 1144)				
No effect/positive effect	807 (70.5)	742 (73.0)	65 (50.8)	**<0.001**
Negative effect	337 (29.5)	274 (27.0)	63 (49.2)	
What effect did the COVID-19 pandemic have on your child’s performance in school or daycare? (n = 1128)				
No effect/positive effect	792 (70.2)	731 (73.0)	61 (48.0)	**<0.001**
Negative effect	336 (29.8)	270 (27.0)	66 (52.0)	
What effect did the COVID-19 pandemic have on your child’s mental health? (n = 1144)				
No effect/positive effect	829 (72.5)	762 (75.0)	67 (52.3)	**<0.001**
Negative effect	315 (27.5)	254 (25.0)	61 (47.7)	

Bolded *p* values for statistical significance (*p* < 0.05).

**Table 4:** Risk Factors for Childhood Underinsurance.

Variable/Level	Adjusted OR *
	(95% CI)
Child’s insurance	
Private	**2.18 (1.28–3.69)**
Public	Reference
PCG’s education status	
≤AA/some college	**3.69 (2.05–6.62)**
≥College graduate	Reference
Getting care is easier/same or harder than 3 years ago?	
Harder	**5.99 (3.58–10.03)**
Easier/stayed the same	Reference
In the past 12 months, did your child’s health suffer because of the inability to pay for medical care?	
Yes	**150.77 (40.55–560.58)**
No	Reference
Child’s age category	
12.0–17.9 years	0.76 (0.37–1.55)
5.0–11.9 years	1.63 (0.96–2.79)
0.0–4.9 years	Reference
Child’s overall health	
Fair/poor	1.76 (0.61–5.11)
Good	1.24 (0.62–2.51)
Very good	1.21 (0.71–2.08)
Excellent	Reference

* Odds ratios are adjusted for all other variables in the model; n = 1051. Of the 1051, 937 (89.2%) are adequately insured and 114 (10.8%) are underinsured. Bolded AOR and CI suggest association between variables. OR, odds ratio; PCG, primary caregiver; AA, Associates of Arts; CI, confidence interval.

## Data Availability

The data presented in this study are available on request from the corresponding author.
